# Long-term renal and cardiovascular risks of tacrolimus in patients with lupus nephritis

**DOI:** 10.1093/ndt/gfae113

**Published:** 2024-05-20

**Authors:** Mieke van Schaik, Obbo W Bredewold, Merel Priester, Wieneke M Michels, Ton J Rabelink, Joris I Rotmans, Y K Onno Teng

**Affiliations:** Center of Expertise for Lupus, Vasculitis and Complement-mediated Systemic disease (LuVaCs), Department of Nephrology, Leiden University Medical Center, Leiden, The Netherlands; Center of Expertise for Lupus, Vasculitis and Complement-mediated Systemic disease (LuVaCs), Department of Nephrology, Leiden University Medical Center, Leiden, The Netherlands; Center of Expertise for Lupus, Vasculitis and Complement-mediated Systemic disease (LuVaCs), Department of Nephrology, Leiden University Medical Center, Leiden, The Netherlands; Department of Nephrology, Leiden University Medical Center, Leiden, The Netherlands; Center of Expertise for Lupus, Vasculitis and Complement-mediated Systemic disease (LuVaCs), Department of Nephrology, Leiden University Medical Center, Leiden, The Netherlands; Department of Nephrology, Leiden University Medical Center, Leiden, The Netherlands; Center of Expertise for Lupus, Vasculitis and Complement-mediated Systemic disease (LuVaCs), Department of Nephrology, Leiden University Medical Center, Leiden, The Netherlands

**Keywords:** calcineurin inhibitors, cardiovascular, chronic renal insufficiency, lupus nephritis, tacrolimus

## Abstract

**Background:**

Despite continuous advancement, treatment of lupus nephritis (LN) remains challenging. Recent guidelines now include a regimen incorporating tacrolimus as a first-line treatment option. Even though tacrolimus is effective in combination with mycophenolate and corticosteroids, concerns remain regarding long-term use, given its association with increased cardiovascular risks including nephrotoxicity, hypertension, dyslipidemia and hyperglycemia in kidney transplant recipients. However, in LN, long-term evaluations and head-to-head comparisons are lacking and thus the safety profile remains ill-defined. We hypothesized that chronic toxicity also occurs in LN patients. Therefore, this study aimed to assess long-term cardiovascular and renal outcomes of tacrolimus in LN patients.

**Methods:**

This observational cohort study examined adult LN patients treated with tacrolimus, assessing renal outcomes, hypertension, diabetes, dyslipidemia, cardiovascular events and the Framingham risk score. The results were compared with a control group of CNI-naïve LN patients.

**Results:**

Of the 219 LN patients in this study, 43 (19.6%) had tacrolimus exposure. Over a median follow-up of 7.1 years, tacrolimus use was associated with significant kidney function decline (6.8 mL/min/1.73 m^2^, versus 0.8 in the control group). The incidence of end-stage kidney disease was similar. Cardiovascular event incidence was equally low in both groups. The 10-year risk of coronary heart disease was lower in the tacrolimus group, primarily due to age differences. HbA1c levels were higher in the tacrolimus group (37.4 mmol/mol) than in controls (33.6 mmol/mol), although the incidence of diabetes was similar. There were no differences in the occurrence of hypertension or dyslipidemia.

**Conclusions:**

Our study demonstrated that tacrolimus exposure was associated with long-term kidney function loss in LN patients. Although cardiovascular risk factors and events were similar to patients never exposed to tacrolimus, there may be an increased risk of developing diabetes. Therefore, our study supports vigilance towards renal adverse effects in LN patients treated with tacrolimus.

## Graphical Abstract

**Graphical Abstract:**
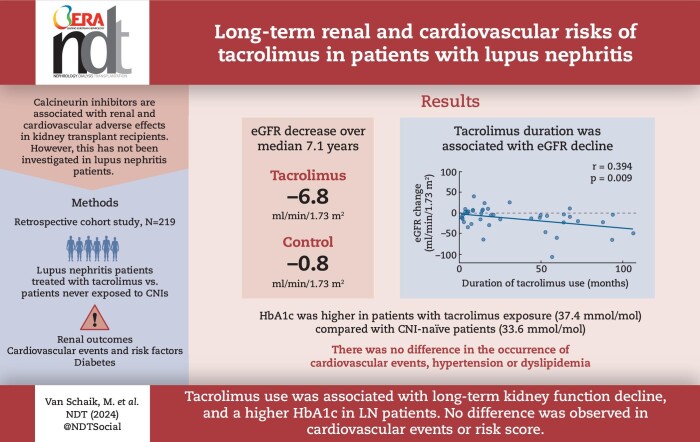

KEY LEARNING POINTS **What was known:**

- Calcineurin inhibitors are now recommended as treatment for active lupus nephritis (LN), in combination with glucocorticoids and mycophenolate.
- Calcineurin inhibitors are associated with renal and cardiovascular adverse effects in kidney transplant recipients. However, this has not been investigated in LN patients.

**This study adds:**

- Tacrolimus use is associated with long-term kidney function decline.
- Cardiovascular adverse events and risk factors are similar in patients using tacrolimus versus patients never exposed to CNIs.
- Tacrolimus may be associated with an increased risk of developing diabetes.

**Potential impact:**

The results of our study advocate caution when prescribing tacrolimus for LN patients, with increased vigilance towards renal adverse effects.

## INTRODUCTION

Lupus nephritis (LN) is a severe and frequent manifestation of systemic lupus erythematosus (SLE), primarily affecting young women. LN follows an unpredictable course in which phases of active disease are alternated by periods of remission, usually induced by potent immunosuppressants. Without proper treatment, LN leads to irreversible kidney damage eventually resulting in end-stage kidney disease (ESKD) [1]. Despite recent advancements with positive impact on kidney and patient survival rates, managing LN remains challenging, and this patient population still faces a diminished life expectancy [2]. Also, with improvements in treatment strategies, causes of morbidity and death in SLE are shifting from disease-related complications to the consequences of prolonged exposure to immunosuppressive medications, such as infections and premature cardiovascular disease [3, 4].

The latest 2023 EULAR guideline includes a regimen incorporating calcineurin inhibitors (CNIs)—tacrolimus or voclosporin—as one of the recommended treatment strategies for active LN [5]. Although CNIs have been less extensively studied in comparison with mycophenolate and cyclophosphamide, tacrolimus, as part of a multitargeted treatment approach, demonstrates equivalent efficacy, and some studies observe superior safety [6–11]. Also, considering that pregnancy poses a frequent dilemma for young women with SLE, clinicians are often prompted to reach out to less teratogenic immunosuppressive agents such as tacrolimus. Tacrolimus finds its origin in solid organ transplantation, and has become the cornerstone of preventing rejection in kidney transplantation [12, 13]. In transplantation, tacrolimus has a well-defined toxicity profile that includes nephrotoxicity, hypertension, dyslipidemia and hyperglycemia [14]. However, data from transplant recipients cannot be directly extrapolated to LN patients due to intrinsic differences in demographics, comorbidities and concomitant treatment.

In LN, all CNIs including cyclosporin, tacrolimus and voclosporin have been studied. Cyclosporin has limited evidence of efficacy in LN, has concerns regarding chronic nephrotoxicity and is inferior to tacrolimus in transplant recipients, thus rendering it a less common choice for LN management [15–19]. Tacrolimus has demonstrated effectiveness in LN across numerous trials, predominantly in Asian LN patients. A major limitation of the reported studies on tacrolimus is the lack of long-term exposure and follow-up to evaluate potential chronic toxicity [20, 21]. Lastly, voclosporin was specifically investigated for LN and is now recommended, in combination with corticosteroids and mycophenolate, as an induction treatment [22, 23]. Although head-to-head comparisons are currently lacking, a meta-analysis suggested that voclosporin may exhibit slightly reduced efficacy, but superiority in terms of infection-related outcomes, when compared with triple therapy containing tacrolimus [24]. However, voclosporin has only recently gained approval as a treatment for active LN and has yet to be integrated in clinical practice.

Altogether, there is a lack of comprehensive long-term data on CNI use in LN patients, and therefore safety concerns remain [5]. Hence, we set out to investigate long-term effects of tacrolimus on kidney function, and cardiovascular risk factors and events in LN patients treated with tacrolimus.

## MATERIALS AND METHODS

### Study design

The study was conducted at the center of expertise on Lupus-, Vasculitis- and Complement-mediated systemic autoimmune diseases (LuVaCs) of the Leiden University Medical Center (LUMC) in the Netherlands. We performed a retrospective observational cohort study, in which we investigated the association between tacrolimus exposure and renal and cardiovascular outcomes. Ethical approval was granted by the local research committee, and passive consent was obtained from all patients.

### Study population

We used an established cohort of 298 LN patients, consisting of all patients with a clinical diagnosis of LN treated in the LUMC between 1 January 2010 and 25 May 2023. Only adult LN patients were eligible for inclusion. Patients were categorized into two groups based on the presence or absence of CNI prescriptions during their treatment period in the LUMC, after which patients who had utilized cyclosporin or topical CNIs, and patients who had <30 days of tacrolimus use were excluded. Patients were also excluded if they had a history of dialysis or kidney transplantation, or if insufficient data were available. This resulted in a tacrolimus group consisting of patients who had experienced systemic tacrolimus exposure, whilst the control group comprised patients who had never been exposed to any form of CNI.

### Follow-up period

For every patient, their first and last hospital visit between 1 January 2010 and 25 May 2023 were respectively designated baseline and follow-up, unless the year of LN diagnosis succeeded the first hospital visit, then the baseline date was adjusted to 1 July of that particular year. Solely for the renal outcomes, patients were excluded once they reached ESKD, with this date serving as the end of their follow-up. All relevant data from this period were then collected, and we compared baseline with follow-up for all outcome measures.

### Data collection

We employed CTcue software, a web-based data-mining tool with text-mining features, to systematically retrieve data from electronic health records in a pseudonymized way (CTcue, v4.8.1, Amsterdam, The Netherlands) [25]. All prescriptions of systemic and topical CNIs for all patients were collected using CTcue, after which we investigated CNI use in clinical documentation of all patients, to fully exclude tacrolimus use in the control group, and obtain the exact period tacrolimus was used in the tacrolimus group. Patient demographics, encompassing age and sex, were collected, along with relevant laboratory data, and medication use spanning the entire follow-up period. Dates of SLE and LN diagnoses and occurrence of cardiovascular events were extracted from clinical documentation and correspondence, as was other relevant information regarding medical history. In the tacrolimus group, the indication for tacrolimus was retrieved from clinical documentation. The reliability and thoroughness of the collected data were ensured by conducting manual review of the health records from randomly chosen patients. In the following section, we further elaborate on the laboratory data, medication use and medical histories that were instrumental in evaluating baseline characteristics and outcome parameters.

### Outcomes

The primary endpoint was change of kidney function. We examined baseline and follow-up measurements of serum creatinine levels to calculate the change in estimated glomerular filtration rate [eGFR; computed using the 2021 Chronic Kidney Disease Epidemiology Collaboration (CKD-EPI) equation]. The secondary renal endpoint was progression to ESKD during follow-up, defined as permanent dialysis, kidney transplantation or a sustained eGFR <15 mL/min/1.73 m^2^, in accordance with KDIGO consensus [26]. Finally, we compared 24-h urine protein excretion at follow-up compared with baseline.

Other secondary endpoints encompassed the incidence of cardiovascular events during follow-up, and cardiovascular risk scores, hypertension, diabetes and dyslipidemia. In more detail, cardiovascular events were defined as the occurrence of a myocardial infarction, cerebrovascular infarction or hemorrhage, transient ischemic attack, coronary interventions such as coronary artery bypass grafting or coronary stent placement, percutaneous angioplasty, or peripheral arterial disease. To calculate the 10-year risk of coronary heart disease, we gathered all relevant predictors required for the Framingham risk score, including total cholesterol, high-density lipoprotein (HDL), presence of hypertension and diabetes, and smoking status [27, 28]. The latter was defined as either active smoking during the follow-up period or within the decade leading up to baseline. The presence of hypertension was defined as antihypertensive medication use, diabetes as antidiabetic medication use and dyslipidemia as statin use. Furthermore, we compared HbA1c measurements, exclusively among patients not taking antidiabetic medications. We assessed statin use and low-density lipoprotein (LDL)-cholesterol levels among patients not taking statins to identify patients with dyslipidemia.

In order to identify and address potential confounders—which we based on clinical knowledge and experience—we calculated differences of all abovementioned outcome parameters and demographics at baseline between the groups, and additionally LN class, duration since SLE and LN diagnosis and total follow-up duration, incidence of cardiovascular events prior to baseline, history of smoking, body mass index (BMI), and the presence of a clinical diagnosis of antiphospholipid syndrome (APS). Also, disease severity may be a major confounding factor. However, disease severity lacks a standardized definition in LN and activity scores are not consistently documented for most patients. Therefore we approximated disease severity by considering the number of times patients were prescribed ≥40 mg of prednisolone (or equivalent) during follow-up as a surrogate parameter.

### Statistics

**Figure 1:**
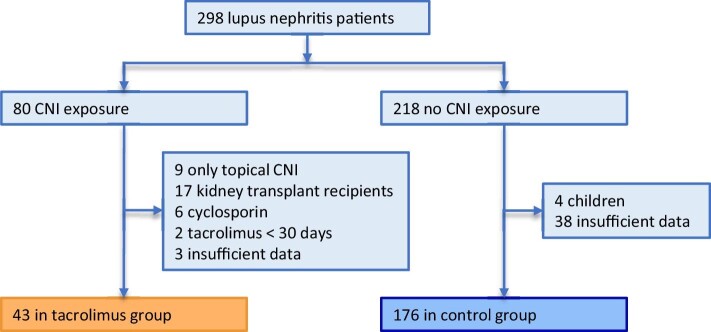
Treatment groups. Flow diagram of the formation of the treatment groups.

Two distinct analyses were performed, a primary analysis, denoted as “by-exposure,” and a subanalysis, termed “on-treatment” (Fig. 1). In the by-exposure analysis, we defined the baseline as the date of the patient's first hospital visit, and the follow-up date as the last visit, as previously described. The advantage of the by-exposure analysis is that it allows for equal follow-up time in both groups. In the on-treatment subanalysis, we defined the baseline as the date tacrolimus treatment was initiated, and follow-up as the date tacrolimus was discontinued. The first measurements were derived from the last observation before the start date of tacrolimus, and the final measurements were derived from the last entry before the stop date of tacrolimus, or exclusion (ESKD). The latter enables a more sensitive analysis regarding tacrolimus use related to outcomes. Since the control group never received tacrolimus, we were unable to apply the aforementioned in this group. Therefore, the baseline and follow-up dates from the by-exposure analysis were used in the control group in the on-treatment analysis as well. It is important to acknowledge that this subanalysis resulted in unequal follow-up durations between the groups.

We presented categorical variables as frequency (%), and continuous variables as median [interquartile range (IQR)]. To analyse differences between baseline and follow-up parameters between the groups, we used Fisher's exact test for comparison of proportions, and the Mann–Whitney U test for comparison of medians. To evaluate the relationship between the duration of tacrolimus use and change in eGFR, we conducted a univariate linear regression analysis. For the primary renal outcome—eGFR change—a multivariate linear regression analysis was performed in which we investigated the effect of tacrolimus on eGFR change after adjustment for confounding variables.

All analyses were carried out using SPSS version 25 (IBM SPSS Software). Missing data were handled by removal of these patients from the respective analyses for which their data was missing. A two-tailed *P*-value of .05 was considered statistically significant.

## RESULTS

### Baseline characteristics

**Figure 2:**
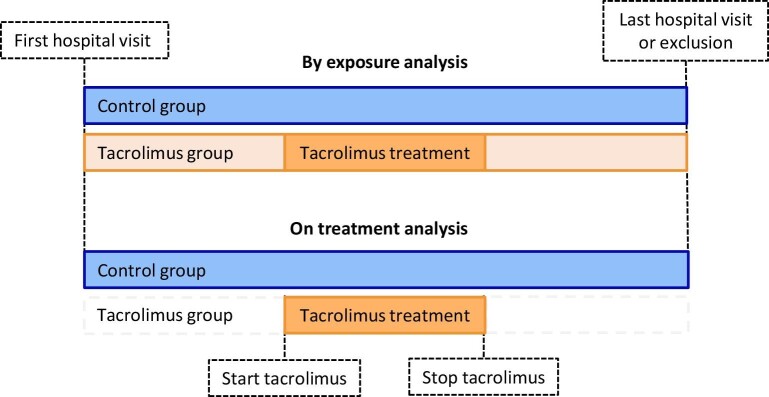
Analyses. Schematic display of the by-exposure and on-treatment analyses.

Of 298 patients in the LN cohort, 80 (26.8%) had CNI exposure, and 218 did not. After exclusion of ineligible patients, 43 (19.6%) were assigned to the tacrolimus group and 176 to the control group (Fig. 2 and Supplementary data, Table S1). Table 1 summarizes the baseline characteristics of both groups. Patients in the tacrolimus group were significantly younger at baseline (31 vs 36 years, *P* = .040) and more often female (90.7% vs 76.1%, *P* = .037) compared with the control group. Median SLE duration was similar among the groups (63.7 vs 53 months, *P* = .596), as was LN duration (14 vs 8.2 months, *P* = .576). The study's follow-up period was median 80.6 (48.4–119.9) months in the tacrolimus group and 88.9 (35.5–178) months in the control group (*P* = .564). Patients were exposed to tacrolimus for a median of 17.7 (6.6–55.2) months, and tacrolimus was indicated for a disease flare in 51.2%, pregnancy (wish) in 32.6% and side effects of previous immunosuppression in 16.3% of patients. Importantly, there was no significant difference in past medical history of cardiovascular events, BMI, smoking history, presence of APS, baseline eGFR, baseline proteinuria, antihypertensive or antidiabetic medication or statin use.

**Table 1:** Baseline characteristics.

Characteristic	Tacrolimus group (*n* = 43)	Control group (*n* = 176)	*P*-value
Age, years	31 (28–39)	36 (30–48)	.040
Sex, female, *n* (%)	39 (90.7)	134 (76.1)	.037
Months since diagnosis			
SLE	63.7 (0–147.9)	53 (0–126.8)	.596
LN	14 (0–105.7)	8.2 (0–86.4)	.576
LN class, *n* (%)			.268
III or IV	20 (46.5)	99 (56.3)	
III + V or IV + V	4 (9.3)	16 (9)	
Pure V	14 (32.6)	33 (18.8)	
Proteinuria, g/day	1.1 (0.4–2.1)	0.9 (0.3–2.3)	.306
BMI, kg/m^2^	24.1 (21.5–27.3)	24.5 (22–28)	.526
eGFR, mL/min/1.73 m^2^	103.2 (63.6–119.4)	91 (60.5–112.1)	.180
History of smoking, *n* (%)	16 (37)	37 (23)	.079
History of cardiovascular events, *n* (%)	2 (4.7)	22 (12.7)	.179
Presence of APS, *n* (%)	8 (18.6)	37 (21)	.835
Medication at baseline, *n* (%)			
Antihypertensive medication	23 (53.5)	71 (40.3)	.126
Antidiabetic medication	1 (2.3)	13 (7.4)	.313
Statins	3 (7)	13 (7.4)	1.000
Follow-up, months	80.6 (48.4–119.9)	88.9 (35.5–178)	.564
Duration of tacrolimus use, months	17.7 (6.6–55.2)	NA	NA
Indication tacrolimus, *n* (%)			
Flare	22 (51.2)	NA	NA
Pregnancy (wish)	14 (32.6)	NA	NA
Side effect	7 (16.3)	NA	NA

Variables are presented as frequency (%), or as median (IQR).

### Kidney outcomes

**Figure 3:**
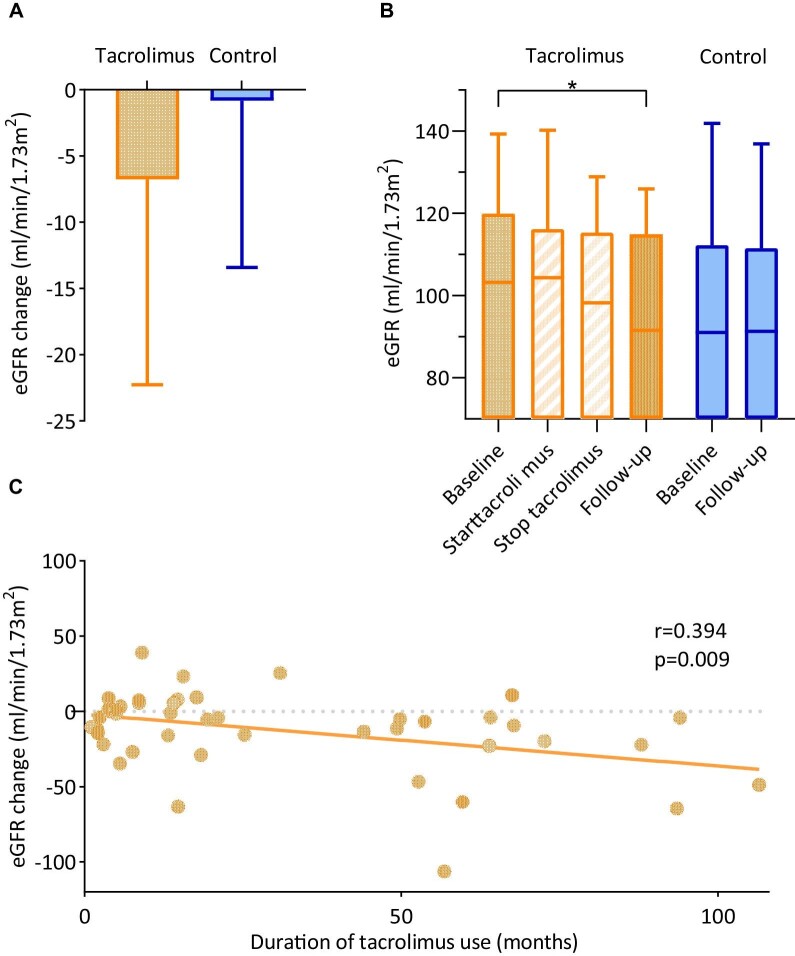
Kidney outcomes. (**A**) eGFR decline (mL/min/1.73 m^2^) in the tacrolimus group compared with the control group. Boxes represent medians and whiskers represent maximum values. (**B**) eGFR change in the tacrolimus group in both the by-exposure as well as the on-treatment analysis, compared with the control group. Boxes represent medians and IQRs, *P < .05. (**C**) Univariate linear regression analysis of the relation between duration of tacrolimus use and eGFR change.

eGFR decline was significantly larger in the tacrolimus group (Table 2): at the end of follow-up, median eGFR change was –6.8 (–22.1 to 2.4) mL/min/1.73 m^2^ in the tacrolimus group, and –0.8 (–13.2 to 9.9) mL/min/1.73 m^2^ in the control group (*P* = .023) compared with baseline (Fig. 3A and B, Table 2). The median eGFR slope was –1.1 mL/min/1.73 m^2^ per year in the tacrolimus group, and –0.1 mL/min/1.73 m^2^ in the control group. A subgroup analysis by indication for tacrolimus treatment demonstrated an eGFR change of –8.6 (–15.9 to 4.6) mL/min/1.73 m^2^ in patients with a disease flare, –4.3 (–22.6 to 2.8) mL/min/1.73 m^2^ in patients with a pregnancy (wish) and –9.4 (–29.2 to –2.4) mL/min/1.73 m^2^ in patients with side effects of previous medications. eGFR decline ≥30% and ≥40% was observed numerically more often in the tacrolimus group (Table 2). There was a difference in neither proteinuria (–0.6 vs –0.5 g/day, *P* = .9), nor in the incidence of ESKD (7% versus 6.8%, *P* = 1.000). Univariate linear regression analysis showed that there was a significant relation between the duration of tacrolimus use and the degree of eGFR decline (r = 0.394, *P* = .009) (Fig. 3C). All three patients in the tacrolimus group that progressed to ESKD did so while being actively on tacrolimus treatment. Two started tacrolimus because of a disease flare, one because of side effects of previous medications. They reached ESKD after 18.4, 49.3 and 56.8 months of active tacrolimus use. The on-treatment subanalysis resulted in similar findings (Fig. 3B and Supplementary data, Table S2). A multivariate linear regression model including tacrolimus use and potentially confounding factors age, sex, disease severity, baseline eGFR and follow-up duration confirmed the relation between tacrolimus use and eGFR decline. Upon adjustment, tacrolimus use was associated with an eGFR decline of 14.7 mL/min/1.73 m^2^ (95% CI –22.5 to –7, *P* < .001). To further evaluate the impact of disease severity, we conducted a sensitivity analysis. This involved stratifying patients for disease severity, comparing those without flare to those with at least one flare during follow-up. In the tacrolimus group, 22 (51.2%) of patients had at least one flare, versus 77 (43.8%) in the control group. Eleven patients had a flare during tacrolimus use, after a median of 26.8 months of use. The sensitivity analysis showed that the effect of tacrolimus was more pronounced in the patients who did not experience a major disease flare (–20 mL/min/1.73 m^2^). This effect persisted irrespective of adjustment for age and sex (Supplementary data, Table S3).

**Table 2:** Kidney outcomes. eGFR, proteinuria and the incidence of ESKD.

Outcome	Tacrolimus group	Control group	*P*-value
eGFR change, mL/min/1.73 m^2^	–6.8 (–22.1 to 2.4)	–0.8 (–13.2 to 9.9)	.023
Indication			
Flare	–8.6 (–15.9 to 4.6)	NA	
Pregnancy (wish)	–4.3 (–22.6 to 2.8)	NA	
Side effect	–9.4 (–29.2 to –2.4)	NA	
eGFR decline ≥30%, *n* (%)	11 (25.6)	27 (16)	.120
eGFR decline ≥40%, *n* (%)	7 (16.3)	21 (12.4)	.449
Proteinuria, g/day	0.3 (0.2–0.8)	0.2 (0.1–0.5)	.076
Proteinuria change, g/day	–0.6 (–1.4 to –0.1)	–0.5 (–1.7 to 0)	.961
ESKD, *n* (%)	3 (7.0)	12 (6.8)	1.000

Variables are presented as frequency (%), or as median (IQR).

### Cardiovascular events and risk score

In this study, 18 cardiovascular events occurred; five events in four patients (9.3%; 17.4/1000 person years) in the tacrolimus group, and 13 events (7.4%; 10/1000 person years) in the control group (*P* = .750) (Table 3). Due to the small number of events, neither evaluation of confounding factors nor stratification for duration of tacrolimus use was performed. Of note, one event in the tacrolimus group occurred during tacrolimus use, 1 year after tacrolimus was started. Two events in one patient occurred 3 and 4 years after discontinuation of tacrolimus, which had been used for 60 days. The remaining two events preceded the initiation of tacrolimus. Fifty percent of patients with cardiovascular events in the tacrolimus group had a diagnosis of APS, versus 38.5% in the control group. The tacrolimus group had a significantly lower Framingham-predicted cardiovascular risk at the end of follow-up compared with the control group (median of 2% vs 3.5%, *P* = .030) (Table 3).

**Table 3:** Cardiovascular events and Framingham risk score.

Outcome	Tacrolimus group	Control group	*P*-value
Cardiovascular events			
MI/CABG/PCI	2	4	
CVA/TIA	2	9	
PAD	1	0	
Total	5	13	
Patients with ≥1 event, *n* (%)	4 (9.3)	13 (7.4)	.750
Framingham risk score			
10-year risk at follow-up, %	2 (1.2–4.3)	3.5 (1.5–10.1)	.030
Age, years	38 (33.5–44.8)	49 (37–58)	
Sex, female, *n* (%)	38 (90.5)	94 (72.9)	
Systolic blood pressure	130.5 (115–140)	125 (115–142)	
Hypertension treatment, *n* (%)	25 (59.5)	70 (54.3)	
Smoking, *n* (%)	4 (9.5)	16 (12.4)	
Diabetes, *n* (%)	2 (4.8)	20 (15.5)	
Total cholesterol	154.5 (127.2–184.3)	151.2 (128.4–173.2)	
HDL	59.7 (43.8–71.5)	56.5 (44.5–73.5)	

Variables are presented as frequency (%), or as median (IQR).

MI, myocardial infarction; CABG, coronary artery bypass graft; PCI, percutaneous coronary intervention; CVA, cerebrovascular accident; TIA, transient ischemic attack; PAD, peripheral arterial disease.

### Hypertension, diabetes and dyslipidemia

The number of patients using antihypertensive medications increased from 23 to 26 (50.5%) in the tacrolimus group and from 71 to 98 (55.7%) in the control group, which was not significantly different (*P* = .610) (Table 4). The number of patients using antidiabetic medications increased from 1 to 2 (4.7%) in the tacrolimus group and from 13 to 22 (12.5%) in the control group (*P* = .179). HbA1c was higher in the tacrolimus group than the control group [37.4 (34.1–41.8) vs 33.6 (33.6–36.7) mmol/mol, *P* = .005]. The number of patients using statins increased from 3 to 6 (14%) in the tacrolimus group and from 13 to 34 (19.3%) in the control group (*P* = .513), and LDL was similar in both groups. Interestingly, in the on-treatment analysis, the number of patients on antihypertensive medications decreased from 23 to 18 (42.9%) in the tacrolimus group, and increased from 71 to 98 (55.7%) in the control group (*P* = .126). No patients started antidiabetic medication during tacrolimus use, and one patient started a statin.

**Table 4:** Cardiovascular outcomes. Hypertension, diabetes and dyslipidemia.

Outcome	Tacrolimus group	Control group	*P*-value
Hypertension			
Antihypertensive medication use, *n* (%)	26 (50.5)	98 (55.7)	.610
Diabetes			
Antidiabetic medication use, *n* (%)	2 (4.7)	22 (12.5)	.179
HbA1c, mmol/mol	37.4 (34.1–41.8)	33.6 (23–36.7)	.005
Dyslipidemia			
Statin use, *n* (%)	6 (14)	34 (19.3)	.513
LDL, mmol/L	2.5 (1.8–2.9)	2.4 (2.1–3)	.826

Variables are presented as frequency (%), or as median (IQR).

## DISCUSSION

This study demonstrates that LN patients exposed to tacrolimus treatment had significant loss of kidney function over time compared with LN patients not exposed to tacrolimus. Although bias by indication was difficult to rule out, tacrolimus appeared independently associated with kidney function decline. Notably, tacrolimus was administered to LN patients who were younger and had better baseline kidney function, and follow-up duration was slightly shorter in this group. Cardiovascular outcomes were similar between both LN groups. Although the higher HbA1c levels in the tacrolimus group may suggest an increased risk for developing diabetes, we could not corroborate this in our cohort.

With the recent approval of voclosporin [29, 30], three CNIs are available for the treatment of LN. Each CNI has a different mechanism of action to inhibit the calcineurin system at the molecular level and can therefore vary with respect to its toxicity profile. For all three, scrutiny remains with respect to canonical CNI-related nephrotoxicity, hypertension and diabetes [31, 32]. Because multiple CNIs are recommended for patients with active LN, the pivotal arguments for clinical decision-making will evolve, aside from availability and accessibility, towards the safety profiles. Therefore, with respect to chronic CNI toxicity profiles, it is indispensable to understand long-term cardiovascular and renal risks as common CNI-related side-effects. Meta-analyses and pharmacodynamics-based models have attempted to compare the CNIs in terms of effectiveness and adverse effects, and this study corroborates that chronic nephrotoxicity from tacrolimus also occurs in LN patients [24, 33].

Several studies have investigated kidney function loss and progression to ESKD over time in LN, documenting an eGFR trajectory of 0 to –3 mL/min/1.73 m^2^ annually—which is particularly pronounced in specific subsets of patients, such as those with class IV proliferative nephritis and those with ongoing disease activity or flares—and an overall 10-year incidence of ESKD of 10%, in accordance with our observations [23, 34–37]. Compared with other LN studies, cardiovascular risk factors were less common in our cohort. A very large cross-sectional cohort study reported hypertension, dyslipidemia and diabetes prevalence rates of 91%, 46% and 28%, respectively [38]. Smaller studies show an incidence of hypertension comparable to our findings [39–41]. Our cohort's cardiovascular event rate was similar to other studies in LN, showing between 10 and 20 events per 1000 person-years [42, 43].

Our study comes with limitations that should be acknowledged. First and foremost, despite our efforts to account for disease severity, the possibility that the observed eGFR decline in the tacrolimus group was influenced by differences in disease severity could not be entirely excluded or fully corrected for. Nevertheless, to counter potential biases arising from such variations we have employed multiple measures, including the assessment of proteinuria, baseline eGFR, LN class and the occurrence of major flares. The surrogate marker utilized to assess the latter, although grounded in clinical experience, complements these measures in minimizing any disparities in disease severity between study groups. It is worth noting, however, that LN patients treated with tacrolimus had a higher baseline eGFR, implying that physicians might have been more inclined to prescribe tacrolimus when patients exhibited better kidney function. Conversely, we observed that during tacrolimus treatment, the number of prescribed antihypertensives decreased in this group, suggesting improved control of proteinuria in often normotensive, young LN patients. Secondly, it is important to acknowledge that the accuracy of the CKD-EPI estimation equation for GFR is less certain and generally underestimated at higher GFR [44, 45]. However, measured GFRs were not available for all patients, and CKD-EPI estimated GFR slope appears to be accurate for identification of progressive kidney function loss [46]. Thirdly, measurements collected at the available time points relative to baseline and follow-up dates might not exclusively reflect disease remission, and transient fluctuations could be present due to variations in disease activity. Fourthly, the absence of an association between tacrolimus use and cardiovascular risk factors may be explained by the older age and higher proportion of males in the control group. Finally, even though the follow-up duration was long, any effect may have been mitigated by the relatively young age of this patient population, and it is conceivable that they will become apparent at a later age.

In conclusion, this study showed a clinically meaningful kidney function decline in LN patients exposed to tacrolimus, its association with treatment duration, and its apparent independence of tacrolimus indication. These observations underscore the necessity of conducting prospective studies in well-balanced cohorts investigating long-term CNI use, especially because calcineurin inhibitors including tacrolimus offer a valuable treatment option for (refractory) LN, during pregnancy and in case of intolerance of other immunosuppressive medications. Thus, our study supports the need for increased vigilance towards tacrolimus treatment, especially in LN patients with an increased risk of developing ESKD.

## Supplementary Material

gfae113_Supplemental_File

## Data Availability

Data will be available upon reasonable request.
