# Supplementary material for: Long-term renal and cardiovascular risks of tacrolimus in patients with lupus nephritis
Source: Nephrol Dial Transplant. 2024 May 20;39(12):2048–57. doi: 10.1093/ndt/gfae113 (PMC11596090; doi:10.1093/ndt/gfae113)
Supplement: gfae113_Supplemental_File [file gfae113_supplemental_file.docx]

**Supplementary tables + table legends**

**Supplementary table 1: Use of immunosuppressive medication.** Variables are presented as frequency (%).

| Medication | Use during follow-up |
| --- | --- |
| Azathioprine | 59 (26.9%) |
| Belimumab | 75 (34.2%) |
| Cyclophosphamide | 30 (13.7%) |
| Lelfunomide | 1 (0.5%) |
| Methotrexate | 14 (6.4%) |
| Mycophenolate | 158 (72.1%) |
| Glucocorticoids | 188 (85.8%) |
| Rituximab/obinutuzumab | 62 (28.3%) |
| Tacrolimus | 43 (19.6%) |

**Supplementary table 2: Kidney outcomes in the on-treatment subanalysis.** Estimated glomerular filtration rate, proteinuria and the incidence of ESKD

| Outcome | Tacrolimus group | Control group | P-value |
| --- | --- | --- | --- |
| *Kidney function* |  |  |  |
| eGFR change, ml/min/1.73m^2^ | -6.9 (-15.1 - 1.2) | -0.8 (-13.2 - 9.9) | 0.059 |
| Proteinuria change, g/day | -0.2 (-0.8 - 0) | -0.5 (-1.7 – 0) | 0.166 |
| ESKD | 3 (7%) | 13 (7.4%) | 1.000 |

**Supplementary table 3. Multiple regression analysis.** Results of a multiple linear regression analysis with adjustment for potential confounding factors age (years), sex (0; male, 1; female), disease severity (number of flares), baseline eGFR (per mL/min/1.73m^2^), and follow-up duration (months). CI; confidence interval. * <0,05, ** <0,01, *** <0,005, **** <0,001.

|  | Crude eGFR change (ml/min/1.73m^2^) | Adjusted eGFR change (ml/min/1.73m^2^) | 95% CI (significance level) |
| --- | --- | --- | --- |
| Tacrolimus use | -10,7 |  | -19,9 - -1,9 (*) |
| Tacrolimus use  + age  + sex |  | -11,5 | -20,6 - -2,4 (*) |
| Tacrolimus use  + age  + sex  + disease severity |  | -12,5 | -21,8 - -3,2 (**) |
| Tacrolimus use  + age  + sex  + disease severity  + baseline eGFR  + follow-up duration |  | -14,7 | -22,5 - -7 (****) |
| Sensitivity analysis |  |  |  |
| *No flares* |  |  |  |
| Tacrolimus use | -17,6 |  | -29 - -6,2 (***) |
| Tacrolimus use  + age  + sex |  | -20 | -31,1 - -9 (****) |
| *1 or more flares* |  |  |  |
| Tacrolimus use | -4,4 |  | -18,2 – 9,5 |
| Tacrolimus use  + age  + sex |  | -4,8 | -18,9 – 9,3 |
